# Premix Membrane Emulsification: Preparation and Stability of Medium-Chain Triglyceride Emulsions with Droplet Sizes below 100 nm

**DOI:** 10.3390/molecules26196029

**Published:** 2021-10-04

**Authors:** Lara El-Hawari, Heike Bunjes

**Affiliations:** 1 Institut für Pharmazeutische Technologie und Biopharmazie, Technische Universität Braunschweig, Mendelssohnstraße 1, D-38106 Braunschweig, Germany; l.el-hawari@tu-braunschweig.de; 2 Zentrum für Pharmaverfahrenstechnik (PVZ), Franz-Liszt-Straße 35a, D-38106 Braunschweig, Germany

**Keywords:** nanoemulsion, colloidal drug carriers, premix membrane emulsification, alumina membrane, Ostwald ripening, sucrose laurate, ultrahydrophobe

## Abstract

Premix membrane emulsification is a promising method for the production of colloidal oil-in-water emulsions as drug carrier systems for intravenous administration. The present study investigated the possibility of preparing medium-chain triglyceride emulsions with a mean particle size below 100 nm and a narrow particle size distribution using sucrose laurate as an emulsifier. To manufacture the emulsions, a coarse pre-emulsion was repeatedly extruded through alumina membranes (Anodisc^™^) of 200 nm, 100 nm and 20 nm nominal pore size. When Anodisc^™^ membranes with 20 nm pore size were employed, nanoemulsions with z-average diameters of about 50 nm to 90 nm and polydispersity indices smaller than 0.08 could be obtained. Particle growth due to Ostwald ripening was observed over 18 weeks of storage. The Ostwald ripening rate linearly depended on the emulsifier concentration and the concentration of free emulsifier, indicating that micelles in the aqueous phase accelerated the Ostwald ripening process. Long-term stability of the nanoemulsions could be achieved by using a minimised emulsifier concentration or by osmotic stabilisation with soybean oil added in a mass ratio of 1:1 to the lipid phase.

## 1. Introduction

Many newly developed drugs have a poor water solubility and need special formulations to be administered to patients. Colloidal lipid emulsions are being intensively studied as carrier systems to address this issue [1,2]. Oil-in-water type nanoemulsions can be regarded as suitable for intravenous administration if they comply with several requirements to avoid undesired effects: a mean particle size smaller than 500 nm, a narrow particle size distribution and the use of biocompatible ingredients [3].

Lipid nanoemulsions for parenteral use are commonly manufactured by high-pressure homogenisation. Recently, premix membrane emulsification (PME) has been established as a promising alternative to produce these systems. In this procedure, a coarse pre-emulsion is repeatedly extruded through a nanoporous membrane, resulting in droplets with sizes in the nanometre range. Pressures of up to only 60 bar are applied in this process, leading to virtually no heat production [4]. The low energy input could be an advantage for thermolabile substances or substances that are sensitive to shear stress. In addition, nanoemulsions can be produced at very small scales (e.g., 0.5 mL) with PME, which is a benefit in the development process of new formulations with highly potent or expensive drugs. High-pressure homogenisation may lead to particle sizes in the lower nanometre range but often yields rather broad particle size distributions [5,6]. In contrast, very narrow particle size distributions can be achieved with PME [7,8].

Most commonly, Shirasu Porous Glass membranes are used in the PME process but various other membrane materials can also be employed [9,10,11]. The majority of the studies using this technique were performed with regard to the production of droplet sizes in the micrometre range [12,13,14,15,16]. It has, however, been shown that it is possible to produce emulsions with droplet diameters smaller than 500 nm with different membrane materials [4,8,17,18,19,20]. The resulting particle sizes are mainly controlled by the pore size of the membranes. Regarding membranes with pores in the micrometre range, the ratio of the mean particle size to the mean pore size increases with decreasing pore size [12,21]. Furthermore, other influencing factors such as good wettability of the membrane with the continuous phase and the composition of the nanoemulsion must be considered. A previous study reported promising particle size results below 100 nm using the Anodisc^™^ 200 nm membrane with medium-chain triglycerides as the disperse phase and sodium dodecyl sulphate as an emulsifier [22]. However, sodium dodecyl sulphate is not appropriate for parenteral formulations because of its cytotoxic potential [23]. Anodisc^™^ membranes are made of alumina and have a higher hydrophilicity than common polymeric membranes. According to the manufacturer, Anodisc^™^ membranes have defined pore sizes, a narrow pore size distribution and are available in 200 nm, 100 nm and 20 nm pore diameter [24].

As an essential property of pharmaceutical preparations, a good stability of the small-sized nanoemulsions against droplet growth should be ensured. Several destabilisation processes can occur in emulsions, e.g., Ostwald ripening and coalescence. Coalescence is the merging of two droplets that appears when the droplets tend to coagulate and the droplet surface is not completely covered with emulsifier [25]. Upon Ostwald ripening, large droplets grow at the expense of smaller ones due to the higher solubility of the lipid in the small droplets. Simultaneously, the particle size distribution narrows, which is a major characteristic of Ostwald ripening and distinguishes it from coalescence [26,27].

The present study addresses the question whether it is possible with PME to obtain nanoemulsions with mean particle sizes smaller than 100 nm and narrow particle size distributions (polydispersity index (PDI) smaller than 0.1) with the perspective of parenteral use. The smaller particle sizes compared to those typically applied for parenteral nutrition enable an extended circulation time in the bloodstream as the droplets are less likely to be recognised and eliminated by the mononuclear phagocyte system. This would be advantageous when using nanoemulsions as drug carrier systems, e.g., for passive tumour targeting [1,2]. Anodisc^™^ membranes were chosen for the PME experiments and medium-chain triglycerides (MCT), which are a component of many commercially available parenteral lipid nanoemulsions, were used as the disperse lipid phase [28]. Sucrose laurate (SL) was applied as an emulsifier based on the promising results of previous studies [22,29]. Although not yet authorised for intravenous formulations, it is considered as a potential pharmaceutically relevant, safe and biodegradable nonionic emulsifier [30]. A further aim was to control the particle size growth caused by Ostwald ripening to obtain long-term stable nanoemulsions.

## 2. Results and Discussion

### 2.1. Membrane Selection and Characterisation

To evaluate which membrane may be suitable for the preparation of nanoemulsions with droplet diameters smaller than 100 nm, an excess of emulsifier (10% SL) was applied for this experiment. Thus, the achievable particle size range for 200 nm, 100 nm and 20 nm pore size Anodisc^™^ membranes could be determined according to earlier approaches of our group [29].

**Figure 1:**
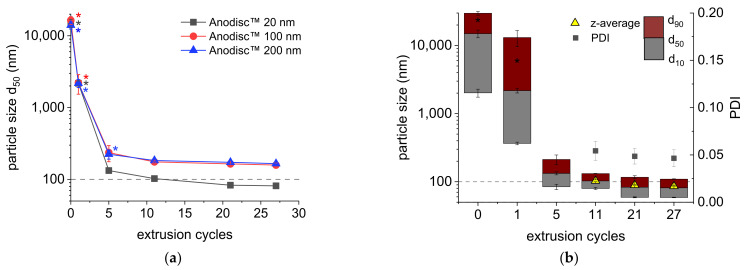
(**a**) Median particle sizes (d_50_) of MCT (10%) nanoemulsions with 10% SL obtained by PME through Anodisc^™^ membranes with 20 nm, 100 nm and 200 nm nominal pore size; (**b**) particle size distribution, z-average diameter and PDI of MCT (10%) nanoemulsions with 10% SL obtained by PME through a 20 nm Anodisc^™^ membrane, dependent on extrusion cycle numbers (flow rate 0.2 mL/s). Mean of 3 independent experiments (*n* = 3) ± standard deviation; asterisks label bimodal particle size distributions.

As expected, the median particle size and size distribution width decreased with increasing number of extrusion cycles for all membranes (Figure 1a). The results were comparable to those of other studies with different 200 nm pore-sized membrane materials [17]. The main decrease was observed over the first five cycles, after which usually monomodal nanoemulsions were obtained. With the 200 nm and 100 nm Anodisc^™^ membranes, only a slight decrease in the d_50_ value could be observed when processing with more than 11 cycles. Median particle sizes of 166 ± 8 nm (200 nm Anodisc^™^) and 158 ± 12 nm (100 nm Anodisc^™^) were reached after 27 cycles, which was far above our aim. PME with the 20 nm Anodisc^™^ membrane led to promising nanoemulsions with a d_50_ of 103 ± 3 nm already after 11 cycles, followed by a further decrease in particle size and size distribution width (Figure 1b). The last cycles achieved smaller progress regarding further particle size reduction, leading to a d_50_ value of 81 ± 1 nm (volume weighted) or a z-average diameter of 86 ± 2 nm (intensity weighted), respectively. The particle size distribution width was remarkably narrow (PDI = 0.046) after 27 cycles. Other than in most previous studies of our group [8,17,18,19,20,22,31], the number of extrusion cycles was extended to 27 in order to achieve the maximum emulsification efficiency [29]. As a conclusion of this experiment, 20 nm Anodisc^™^ membranes were appropriate to produce nanoemulsions in the desired particle size range with PME, while 100 nm and 200 nm Anodisc^™^ membranes were not.

**Figure 2:**
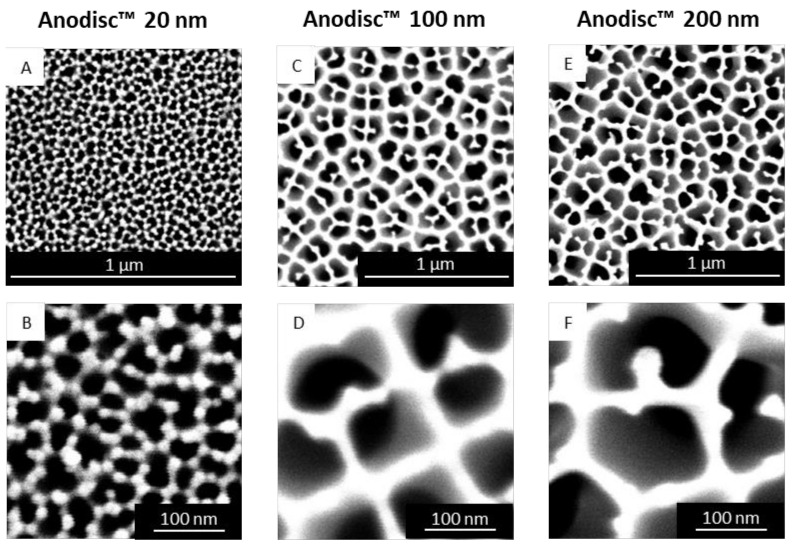
SEM images of representative Anodisc^™^ membranes of 20 nm (**A**,**B**), 100 nm (**C**,**D**) and 200 nm nominal pore size (**E**,**F**) in different magnifications (pores appear dark in the images).

To explain the particle size results obtained, we focused on the structure and pore size of the membranes using scanning electron microscopy (SEM). The Anodisc^™^ membranes showed quite uniformly shaped pores with honeycomb-like structure and high porosity (Figure 2). According to the manufacturer’s specification, the 100 nm Anodisc^™^ and 20 nm Anodisc^™^ are asymmetric membranes with the nominal pore size on the upper side and 200 nm pores on the bottom side. The active layer is less than 1 µm in thickness [32]. When the maximum Feret diameters (Feret_max_) were calculated from the SEM images of each membrane type, obvious differences were detected compared to the nominal pore size of the membranes (Table 1). This may at least be partially due to differences in the measurement method and the specifying values. Moreover, the range of specification for Anodisc^™^ 20 nm membranes is rather broad (pore sizes from 20 nm to 70 nm) due to the complex manufacturing process. The particle sizes resulting from PME corresponded very well to the Feret_max_ pore diameters of the respective membrane but not to the nominal pore sizes as given by the manufacturer. The d_50_ values obtained from the emulsions were never smaller than the Feret_max_ diameter of the pores (with consideration of the standard deviations). The values for the Feret_max_ diameters demonstrated considerable standard deviations and thus pointed to pore size distributions that are not absolutely homogeneous. However, this is not expected to cause major negative effects in PME due to the processing by repeated extrusion [21].

The particle size (d_50_) to pore size (d_p_) ratio may allow conclusions about the emulsification efficiency (Table 1). The ratio was estimated with the nominal pore size as well as with the calculated Feret_max_ values. The d_50_ to d_p_ ratio based on the nominal pore sizes suggested a decreasing emulsification efficiency with decreasing pore size (d_50_/d_p_ = 0.83 to 4.05). However, regarding the ratio calculated on the basis of the Feret_max_ pore sizes, this conclusion was not confirmed and the emulsification efficiency appeared to be similar for all membrane pore sizes (d_50_/d_p_ = 1.1 to 0.95). No change in the emulsification efficiency was observed by Alliod et al. for Shirasu Porous Glass membranes in the nanometre pore size range, which differs from the increasing d_50_/d_p_ ratio with decreasing pore size observed in previous studies carried out with emulsions in the micrometre range [4,12,21]. Different mechanisms may be responsible for the droplet break-up in the nanometre range compared to the micrometre range. Overall, this experiment confirmed that the resulting sizes of the nanodroplets were controlled by the pore size of the Anodisc^™^ membranes with an efficient droplet break-up leading to d_50_/d_p_ values around 1. 

### 2.2. Determination of Minimum Required Emulsifier Concentration

**Figure 3:**
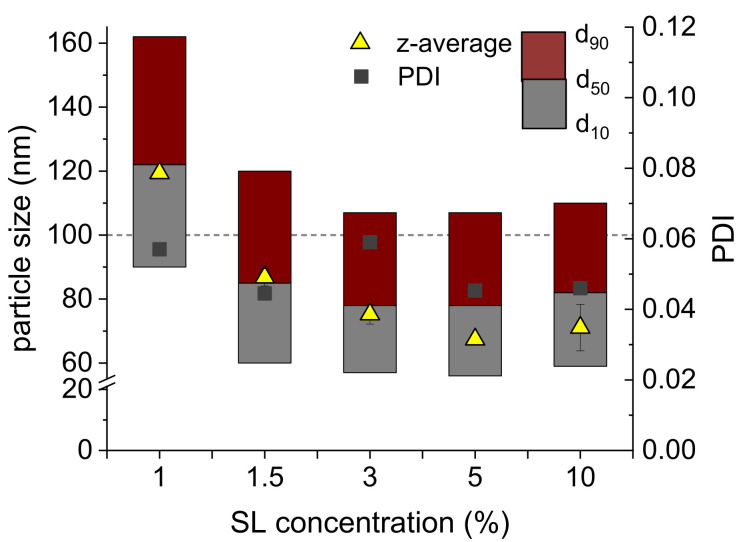
Particle size distributions (d_10_, d_50_ and d_90_ values, *n* = 1), z-average diameter and PDI (*n* = 2) of 10% MCT nanoemulsions prepared by PME through Anodisc^™^ 20 nm membranes (0.2 mL/s) dependent on the SL concentration.

In the emulsification experiments described above, a very high emulsifier concentration was used to avoid any effects on the resulting particle size due to a limited availability of emulsifier molecules for stabilising the droplets. In order to obtain information on the required amount of emulsifier, nanoemulsions were prepared with decreasing SL concentrations (10–1%) to determine the minimum emulsifier concentration (MEC). The MEC was defined as the minimal concentration at which nanoemulsions with a particle diameter below 100 nm and PDI below 0.1 can be produced. A decreasing SL concentration resulted in an increasing particle size (d_50_ = 78–85 nm, z-average diameter = 71–87 nm) but a similar particle size distribution width (PDI = 0.046–0.058) down to 1.5% SL (Figure 3). In contrast, when using just 1% SL, a significant increase in the particle size to above 100 nm was observed (d_50_ = 122 nm, z-average diameter = 119 nm). The particle size distribution width remained unchanged but the nanoemulsion no longer fulfilled the defined requirements. During emulsification, new interfaces are generated due to droplet break up and have to be covered with the emulsifier to stabilise the system. As 1% SL was probably not sufficient to cover the large droplet interfacial area, rapid growth processes by coalescence occurred. At a concentration of 1.5% SL, enough emulsifier was available to stabilise a nanoemulsion with a d_50_ of 85 nm and a narrow particle size distribution (PDI = 0.052). Therefore, 1.5% SL was set as MEC to be used in further investigations.

Theoretically, there should be no free SL molecules left in the aqueous phase of a nanoemulsion prepared with the MEC. To confirm the determined MEC, the concentration of free SL was measured by the Vivaspin method (Section 3.7). In fact, no free SL could be detected refractometrically in the aqueous phase of several nanoemulsions prepared with 1.5% SL (*n* = 4).

### 2.3. Influence of Emulsifier Concentration on Emulsion Stability

**Figure 4:**
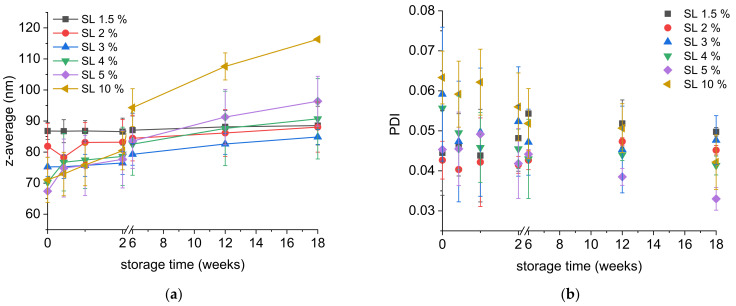
(**a**) Z-average and (**b**) PDI of MCT (10%) nanoemulsions stabilised with 1.5% to 10% SL plotted against the storage time. Nanoemulsions were produced by PME with Anodisc^™^ 20 nm membranes (0.2 mL/s). Mean of 2 independent experiments (*n* = 2) ± standard deviation.

The influence of the SL concentration on the stability of nanoemulsions was determined applying SL concentrations increasing from the MEC up to 10%. In the experiments, the particle sizes of the nanoemulsions measured directly after preparation decreased with increasing SL concentration (Figure 4a). The PDI remained at low values between 0.07 and 0.04 independent of the SL concentration (Figure 4b). At higher emulsifier concentrations, emulsifier molecules are more readily available during the emulsification process, can adsorb more quickly at the new interfaces and lead to smaller particle sizes [12]. Additional effects on the droplet break-up may be caused by the interfacial tension between aqueous and lipid phase and the viscosity of the aqueous phase (increasing with higher SL concentration) in this experiment. According to literature reports, a low interfacial tension and a higher viscosity of the aqueous phase led to a thicker lubrication layer in the membrane pores. As a consequence, the lipid droplets passing the membrane were elongated to a higher extent, thus resulting in smaller droplets [12,33]. 

Upon storage, all nanoemulsions, except for those containing 1.5% SL, exhibited a particle size growth represented by increasing z-average values (Figure 4a). The increase in the z-average diameter of the emulsions containing 1.5% SL remained in the range of the standard deviation (from 87 to 89 nm over 18 weeks). With more SL used for stabilisation, larger particle sizes were detected over time. Nevertheless, all nanoemulsions had retained z-average values below 100 nm at the end of the storage time, except for the emulsion stabilised with 10% SL that reached a z-average of 116 nm. In addition, there was a trend towards decreasing PDI values during storage, pointing to Ostwald ripening as the main growth mechanism (Figure 4b).

**Figure 5:**
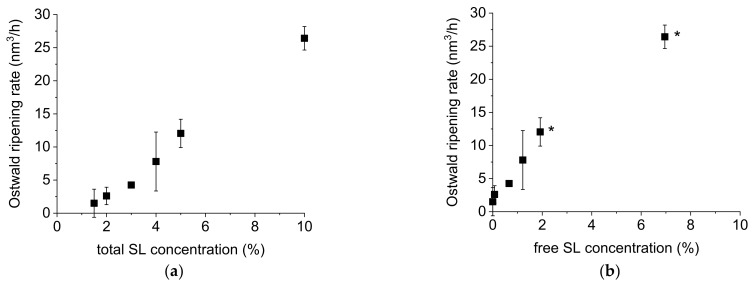
(**a**) Calculated Ostwald ripening rates of MCT nanoemulsions as a function of the applied total SL concentration (mean ± standard deviation, *n* = 2) and (**b**) as a function of the free SL concentration (mean ± standard deviation; determination of free SL concentration *n* = 1; asterisks label values affected by the presence of lipid droplets in the filtrate).

The Ostwald ripening rates were calculated and ranged from 0 nm^3^/h (SL 1.5%) to 26 nm^3^/h (SL 10%). It is already known that nonionic emulsifiers can increase the rate of Ostwald ripening in emulsions and different mechanisms of lipid transport through the continuous phase have been discussed [34,35,36,37]. Compared to studies on alkane emulsions (chain length C10 to C13) stabilised with nonionic surfactants, the Ostwald ripening rates observed in our study were two to five orders of magnitude lower [35,36,38,39]. Very few data are available concerning Ostwald ripening in MCT emulsions, but these are in better agreement with our results (one to three orders lower) [40]. A probable reason for the low Ostwald ripening rates observed here is the very narrow particle size distribution already after preparation by PME. Ostwald ripening is a self-limiting process and stops when all droplets have the same size and thus the same Laplace pressure. In our case, the driving forces for Ostwald ripening are low because of the small differences in Laplace pressure of the droplets. Other reasons could be differences in the emulsifiers used, the type and content of the lipid phase or the experimental setup. A linear relationship was observed between the Ostwald ripening rate and the SL concentration (Figure 5a). This illustrates the strong influence of the emulsifier concentration on the ripening process and the importance of keeping the SL concentration as low as possible (at the MEC) to obtain long-term stable nanoemulsions.

A disadvantage of the current experimental design is that the Ostwald ripening rates may be affected by the different initial particle sizes, which is likely to have an influence on the results. The increase in the radius, used for calculation of Ostwald ripening rates, of small droplets is higher than that of larger droplets considering the same migrated volume of lipid. In addition, the solubility of the lipid droplets is inversely proportional to the droplet size [41]. This aspect can lead to a potential overinterpretation of the increasing Ostwald ripening rates due to higher SL concentrations.

Measuring the concentration of free SL might provide more detailed information about the transport mechanisms responsible for Ostwald ripening. The concentration of free SL was determined with the Vivaspin method (Section 3.7) for all nanoemulsions directly after PME. SL is a rather small emulsifier molecule (M = 524.6 g/mol) and the z-average diameter of SL micelles, determined from a 1% solution in bidistilled water by PCS, was 7 nm ± 0 (*n* = 6). Due to the high MWCO (300 kDa) of the Vivaspin^®^ filter tubes, the SL monomers and the micelles were small enough to pass the filter membrane while lipid droplets were retained. As expected, the concentration of free SL increased with higher SL concentration employed. Above the MEC, the droplet interfaces were sufficiently covered with SL molecules to stabilise the droplets. Therefore, free SL was available in the aqueous phase and when its concentration exceeded the critical micelle concentration (CMC), micelles were formed. The CMC of SL was determined as 0.015% (mean of three independent measurements) and was exceeded by the concentration of free SL in all nanoemulsions prepared with concentrations above the MEC. This implies that the concentration of micelles increased with increasing free SL concentration. Our preliminary data showed a linear relationship between the Ostwald ripening rates and the concentration of free SL in the range of low free SL concentrations (Figure 5b). Higher values were affected by the presence of small lipid droplets in the filtrate and therefore cannot be included reliably in this consideration.

Based on the Lifshitz–Slyozov–Wagner theory, the main mechanism of lipid transport through the continuous phase is diffusion [42,43]. Most experimentally determined Ostwald ripening rates did not comply with values expected from theoretical considerations. Therefore, additional mechanisms were discussed. A possibility is the additional lipid transport by micelles, with the exact mechanisms being still unclear [34,38]. Three hypotheses of lipid uptake and release by micelles were formulated by Kabalnov and discussed and reviewed in other studies [44]. The first hypothesis suggests a fusion–fission mechanism enabling a direct lipid uptake from droplets, assuming that micelles collide with the droplets. This hypothesis was rejected due to electrostatic repulsion effects between droplets and micelles [45]. Moreover, Ariyaprakai and Dungan observed no retardation in ripening processes when the chain lengths of nonionic emulsifier headgroups were increased and thereby disproved the first hypothesis [36]. The second and third hypotheses assume the uptake and release of dissolved lipid from the aqueous phase with different speed. If the micelles are in equilibrium with the dissolved lipid in the aqueous phase, the lipid can be rapidly taken up and released (second hypothesis). However, the diffusion of micelles is relatively fast compared with the time needed for the uptake of the lipid [44]. This led to hypothesis three, according to which the micelles are not in equilibrium with the aqueous phase and the uptake and release of lipid is slow. While the first and second hypotheses predict a direct dependence of the Ostwald ripening rate on the micelle concentration, the third does not [45]. In general, the diffusion speed of lipid-loaded micelles is slower than that of dissolved lipid molecules. Nevertheless, a concentration gradient between loaded micelles and empty micelles could occur, which supports the diffusion of loaded micelles away from the droplets [46]. In our study, the nanoemulsion with 1.5% SL (MEC) effectively exhibited no Ostwald ripening, whereas higher SL concentrations led to increasing Ostwald ripening rates. The results support the second hypothesis of Kabalnov where Ostwald ripening depends on the concentration of micelles involved in the lipid transport through the aqueous phase. Nevertheless, further work is necessary to investigate the transport in SL micelles and the underlying mechanisms. Still, this experiment clearly reveals that the concentration of SL, especially the concentration of free SL, is an important factor and should be minimised in order to achieve the long-term stability of nanoemulsions that are prone to Ostwald ripening.

### 2.4. Osmotic Stabilisation

A different way to achieve small-sized nanoemulsions with higher long-term stability is to incorporate more hydrophobic molecules than the actual lipid, so-called ultrahydrophobes [47], into the lipid phase before PME. Previous studies have shown that Ostwald ripening was decelerated after the addition of ultrahydrophobes [37,48,49,50]. In theory, due to the addition of the ultrahydrophobe, the osmotic pressure in the droplets rises and exceeds the Laplace pressure. Thus, the driving force to dissolve the emulsion droplet is no longer present and Ostwald ripening is slowed down [51]. In practice, the Laplace pressure is often not reached, but the difference between the Laplace pressure and the osmotic pressure is balanced in all emulsion droplets and therefore, the desired effect nevertheless occurs [52]. 

Nanoemulsions made with MCT, a lipid with non-negligible water solubility, are susceptible to Ostwald ripening. Soybean oil, a long-chain triglyceride, is an uncritical ultrahydrophobe with regard to later parenteral administration to humans [3]. The very low water solubility of soybean oil should osmotically stabilise the emulsions and, after an initial increase in particle sizes, should lead to a termination of Ostwald ripening. According to Landfester et al., the type of ultrahydrophobe is irrelevant but the molar ratio between the ultrahydrophobe and the lipid must be at least 1:250 to attain a sufficient osmotic pressure [47]. For the experiments with SL-stabilised MCT emulsions, the composition of the lipid phase was chosen in consideration with that ratio, except for the mass ratio 1:199 corresponding to the molar ratio 1:328. Nanoemulsions containing 5% SL showed a high Ostwald ripening rate in the experiment described above (Section 2.3) and the z-average diameter increased by 30 nm over 18 weeks. Therefore, this SL concentration was selected for osmotic stabilisation experiments. 

**Figure 6:**
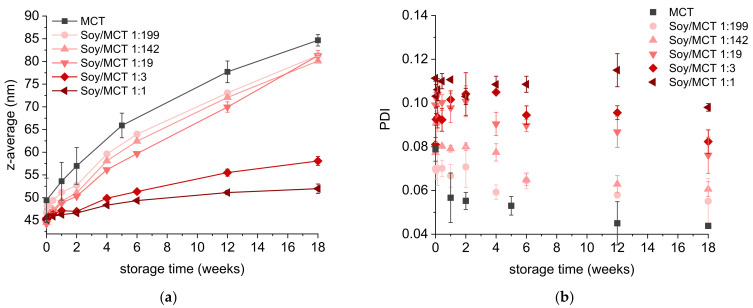
(**a**) Z-average diameter and (**b**) PDI of nanoemulsions with various mass ratios of soybean oil/MCT or just MCT and 5% SL as emulsifier as a function of storage time. Nanoemulsions were produced by PME with Anodisc^™^ 20 nm membranes (0.2 mL/s). Mean of 3 independent experiments (*n* = 3) ± standard deviation.

The particle sizes of all osmotically stabilised nanoemulsions were in the same range independent of the soybean oil/MCT ratio and the particle size distribution was quite narrow with PDI values between 0.07 and 0.11 (Figure 6a,b). The smaller particle sizes (below 50 nm) than obtained in the former experiments (Section 2.1, Section 2.2 and Section 2.3) were caused by using a different batch of Anodisc^™^ 20 nm membranes for PME. In comparison to the MCT nanoemulsions, the osmotically stabilised nanoemulsions displayed less Ostwald ripening. Using 50% ultrahydrophobe (m/m) (sample name Soy/MCT 1:1), the particle size growth over 18 weeks was limited to 15% (referred to the initial particle size) which was 4.6 times less than observed in an MCT nanoemulsion. A stagnation of the particle size growth was not detected until the end of the experiment but should theoretically occur. The decreasing PDI values, observed for most emulsions, confirmed Ostwald ripening as the major growth mechanism (Figure 6b).

**Figure 7:**
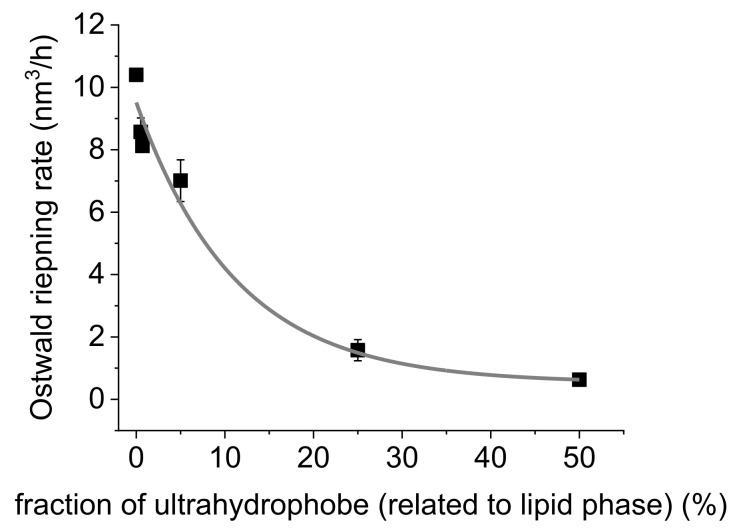
Ostwald ripening rates of osmotically stabilised MCT nanoemulsions as a function of the mass fraction of ultrahydrophobe (soybean oil). The mean of the values was fitted with an exponential function (R-square = 0.9998). Mean of 3 independent experiments (*n* = 3) ± standard deviation.

The more ultrahydrophobe was used, the better was the osmotic stabilisation effect. This resulted in an exponential relationship between the added mass fraction of ultrahydrophobe and the calculated Ostwald ripening rates (Figure 7). A positive effect was already observed with smaller amounts of ultrahydrophobe (1:328 molar ratio) than in the experiments by Landfester et al. [47]. Regardless, the results suggest using 50% ultrahydrophobe to achieve a desirable effect. Increasing the fraction of ultrahydrophobe even further would not provide a large improvement because of the exponential relationship. In addition, the physicochemical characteristics of the lipid phase, e.g., the solubilising capacity for potential drugs, would change significantly using longer-chain triglycerides as ultrahydrophobes [53,54]. In conclusion, osmotic stabilisation is very effective for MCT nanoemulsions containing an excess of the emulsifier SL. In contrast, there was no improvement for nanoemulsions stabilised with the MEC (data not shown), in agreement with the findings of Han et al. [37].

## 3. Materials and Methods

### 3.1. Nanoemulsion Preparation

The nanoemulsions consisted of 10% Miglyol^®^ 812 (MCT) (Caesar & Loretz GmbH, Hilden, Germany) as the lipid phase and various concentrations (1–10%) sucrose laurate (SL) (Surfhope SE Pharma D-1216, donated by Mitsubishi-Chemical Foods Corporation, Tokyo, Japan) in double distilled water, preserved with 0.05% sodium azide (Carl Roth, Karlsruhe, Germany), as the aqueous phase (all percentages refer to the overall emulsion). The two phases were dispersed with an T25 digital ULTRA-TURRAX^®^ (IKA^®^-Werke GmbH & CO. KG, Staufen, Germany) with an S25N-10G device at 10000 rpm for 2 min, leading to a coarse premix. This premix was transferred to the sample container of an instrumented small scale extruder [17]. The extruder is based on a 10 mL high-pressure syringe pump (neMESYS, Cetoni GmbH, Korbußen, Germany) connected to a membrane holder and a computer to control the flow rate and pressure. The membrane holder contains a support screen of stainless steel (pore size 3 mm), a sinter disc (pore size 2 µm) and a drain disc (Whatman^®^ drain disc, Cytiva, Marlborough, CT, USA) underneath the membrane to prevent membrane rupture. Anodisc^™^ membranes (Whatman^®^ Anodisc^™^ 47 with support ring, d_active_ = 41 mm, Cytiva, Marlborough, CT, USA) with nominal pore sizes of 200 nm, 100 nm and 20 nm were used for the extrusion process.

The premix was extruded 27 times through the disposable Anodisc^™^ membranes with a constant flow rate of 0.2 mL/s. Finally, the lipid nanoemulsions were filled into glass vials and stored under nitrogen at 20 °C for 18 weeks.

For the first experiments, regarding the membrane selection (Section 2.1), Anodisc^™^ membranes with all three pore sizes were used and samples of the nanoemulsions were taken after extrusion cycles 1, 5, 11, 21 and 27 for particle size measurement. In the following experiments, the premix was extruded 27 cycles through Anodisc^™^ membranes with 20 nm pore size (several membrane batches were used).

### 3.2. Osmotic Stabilisation of Nanoemulsions

Osmotic stabilisation experiments were carried out with nanoemulsions containing 5% SL. Soybean oil (Caesar & Loretz, Hilden, Germany) was selected as the ultrahydrophobe. The lipid phase (10%) of the nanoemulsions consisted of the mass ratios 1:199, 1:142, 1:19, 1:3 and 1:1 of ultrahydrophobe:MCT corresponding to the molar ratios of approximately 1:328, 1:250, 1:31, 1:5 and 1:2, respectively (M_MCT_ = 561 g/mol, M_soybean oil_ = 925 g/mol). After nanoemulsion preparation (as described in Section 3.1) with a 20 nm Anodisc™ membrane, the samples were stored under nitrogen at 20 °C for 18 weeks.

### 3.3. Particle Size Measurements

The particle sizes of the nanoemulsions were measured using laser diffraction with polarisation intensity differential scattering technology (LD-PIDS) (LS 13320, Beckman Coulter GmbH, Krefeld, Germany). After adequate dilution, three measurements of 90 s each were carried out and averaged to calculate the volume median diameter (d_50_ value), d_10_ and d_90_ values. Additionally, particle sizes (z-average diameter) and particle size distribution widths (expressed as polydispersity index (PDI)) of the nanoemulsions were measured by photon correlation spectroscopy (PCS) (Zetasizer Nano ZS, Malvern Instruments, Worcestershire, UK) at 25 °C and a backscattering angle of 173°. The samples were diluted to an attenuator of 6–8 and the average of three measurements of 3 min after 5 min equilibration time was determined. 

Two complementary particle sizing techniques were used to avoid the limitations of measurement quality of the LD-PIDS technique in the lower nanometre range and of PCS for broad particle size distributions. Due to unpredictable particle size results, the particle size determination during the experiments in Section 2.1 and Section 2.2 were carried out with LD-PIDS as well as PCS; all other values were determined by PCS only.

### 3.4. Determination of Ostwald Ripening Rates

The calculation of the Ostwald ripening rate *ω* was based on the Lifshitz–Slyozov–Wagner theory [42,43], according to which Ostwald ripening is a diffusion-controlled process with the cube of the number average droplet radius ${\overset{-}{r}}^{3}$ being proportional to the time *t* [44].

(1) $$\omega =\frac{{\overset{-}{\mathrm{dr}}}^{3}}{\mathrm{dt}}$$

The number average radius was determined from PCS measurements applying the Mie theory (n_lipid_ = 1.45, n_imaginary_ = 0.01, n_continuous_ = 1.33). All measurement values within the 18 weeks of storage were plotted against the time and the Ostwald ripening rate ω was determined from the slope of the linear fit.

### 3.5. Scanning Electron Microscopy

To investigate the membrane structure and pore size, scanning electron microscopy (SEM) images of the upper side of the Anodisc^™^ membranes were taken with a Helios G4 CX microscope (FEI, Hillsboro, OR, USA) and an Everhart–Thornley detector. Before investigation, small pieces were broken out of the membranes and sputtered with 6 nm platinum (working distance 50 mm) in a high-vacuum coater (Leica EM ACE600, Leica Microsystems GmbH, Wetzlar, Germany). The images were taken with a voltage of 10 kV at a distance of 4 to 6 mm and different magnifications.

The size of all membrane pores of one representative image (per membrane) were measured and averaged with the software ImageJ. Images of 25,000× magnification (200 nm and 100 nm Anodisc^™^) and 120,000× magnification (20 nm Anodisc^™^) were used for this analysis. The software calculated the maximum Feret diameter (Feret_max_) measuring the maximum distance between two parallel tangents applied to the pore (at any angle).

### 3.6. Determination of Critical Micelle Concentration

The critical micelle concentration of SL (CMC) was measured by the surface tension method with a tensiometer (K100, KRÜSS GmbH, Hamburg, Germany) equipped with a Wilhelmy plate at 25 °C. A solution of SL was stepwise added to double distilled water. After each addition, the sample was stirred for 2 min followed by a 2 min waiting interval. Then, 20 measurements in 180 s were recorded and the last five values (in equilibrium state) were averaged. The averaged values were plotted on a logarithmic scale against the surfactant concentration and the CMC was determined at the intersection of the linear fits. All measurements were performed in triplicate.

### 3.7. Determination of the Concentration of Free SL (Vivaspin Method)

The concentration of free SL was defined as the concentration of emulsifier that is not bound to any interface and is available as monomer or micelle in the aqueous phase. A part of the aqueous phase was separated from the emulsion with the aid of Vivaspin^®^ 6 tubes (MWCO 300 kDa, Sartorius, Göttingen, Germany). Prior to the sample preparation, the Vivaspin^®^ tubes were washed three times with bidistilled water and dried to remove substances from the manufacturing process. The concentration of free SL was measured after filtering 5 mL of the respective nanoemulsion through Vivaspin^®^ tubes with the aid of centrifugation for about 30 min (force 500× *g*, Allegra^™^ 64R, Beckman Coulter GmbH, Krefeld, Germany) or until 300 µL filtrate had emerged. The filtrate was collected in the bottom part of the tube. The refraction index of the filtrate was measured (Abbemat WR, Anton Paar, Graz, Austria) and the concentration of free SL was determined with the aid of a calibration curve. The concentration of free SL always refers to the total emulsion.

A control experiment was carried out where no lipid was present. A recovery rate of 96% SL was achieved with a 2% SL solution as well as a 10% SL solution. Based on this high recovery rate, no mathematical corrections were implemented. It was verified before and after centrifugation that no instabilities of the emulsion occurred during the experiment, defined as a maximum deviation of 5% between the determined z-average diameters and PDI values.

## 4. Conclusions

MCT nanoemulsions (10%) with a mean particle diameter below 100 nm can successfully be produced by using premix membrane emulsification with Anodisc^™^ 20 nm membranes. The method enables a low-energy production of mean particle sizes down to 49 nm with very narrow particle size distributions dependent on the concentration of the emulsifier SL. The resulting particle sizes are strongly controlled by the Feret_max_ of the pore sizes of the Anodisc^™^ membranes.

Long-term stability of the nanoemulsions can be achieved by using sufficient but preferably low emulsifier concentrations, optimally the MEC. Above the MEC, micelles are present in the aqueous phase, promoting Ostwald ripening effects in a concentration-dependent manner. Further work is required to understand the mechanism of how nonionic emulsifiers influence Ostwald ripening and what are the rate-determining steps.

At SL concentrations above the MEC, osmotic stabilisation is a successful approach to slow down Ostwald ripening. A significant fraction of ultrahydrophobe is, however, required to achieve a good effect in MCT emulsions with particle sizes below 100 nm.

## Figures and Tables

**Table 1:** Feret_max_ pore diameters calculated from SEM images of Anodisc^™^ membranes, resulting particle sizes of the MCT emulsions (10% SL) and particle size to pore size ratios (d_50_/d_p_) related to either Feret_max_ diameter or to the nominal pore size of the membranes ± standard deviation (*n* = number of measured pores).

Anodisc™ Membrane Type	Feret_max_ ± SD [nm]	Resulting Particle Size d_50_ ± SD [nm] ^1^	d_50_/d_p_ Feret_max_ Pore Size	d_50_/d_p_ Nominal Pore Size
200 nm 100 nm 20 nm	156 ± 32 *n* = 1960 144 ± 33 *n* = 2024 86 ± 40 *n* = 283	166 ± 8	1.06	0.83
		158 ± 12	1.10	1.58
		81 ± 1	0.95	4.05

^1^ after 27 manufacturing cycles, *n* = 3.

## Data Availability

The data are contained within the article.

## Sample Availability

Samples of the lipid carrier systems are not available from the authors.
